# *Chromulinavorax destructans*, a pathogen of microzooplankton that provides a window into the enigmatic candidate phylum Dependentiae

**DOI:** 10.1371/journal.ppat.1007801

**Published:** 2019-05-31

**Authors:** Christoph M. Deeg, Matthias M. Zimmer, Emma E. George, Filip Husnik, Patrick J. Keeling, Curtis A. Suttle

**Affiliations:** 1 Department of Microbiology and Immunology, University of British Columbia, Vancouver, Canada; 2 Department of Earth, Ocean and Atmospheric Sciences, University of British Columbia, Vancouver, Canada; 3 Department of Botany, University of British Columbia, Vancouver, Canada; 4 Institute for the Oceans and Fisheries, University of British Columbia, Vancouver, Canada; Pennsylvania State University, UNITED STATES

## Abstract

Members of the major candidate phylum Dependentiae (a.k.a. TM6) are widespread across diverse environments from showerheads to peat bogs; yet, with the exception of two isolates infecting amoebae, they are only known from metagenomic data. The limited knowledge of their biology indicates that they have a long evolutionary history of parasitism. Here, we present *Chromulinavorax destructans* (Strain SeV1) the first isolate of this phylum to infect a representative from a widespread and ecologically significant group of heterotrophic flagellates, the microzooplankter *Spumella elongata* (Strain CCAP 955/1). *Chromulinavorax destructans* has a reduced 1.2 Mb genome that is so specialized for infection that it shows no evidence of complete metabolic pathways, but encodes an extensive transporter system for importing nutrients and energy in the form of ATP from the host. Its replication causes extensive reorganization and expansion of the mitochondrion, effectively surrounding the pathogen, consistent with its dependency on the host for energy. Nearly half (44%) of the inferred proteins contain signal sequences for secretion, including many without recognizable similarity to proteins of known function, as well as 98 copies of proteins with an ankyrin-repeat domain; ankyrin-repeats are known effectors of host modulation, suggesting the presence of an extensive host-manipulation apparatus. These observations help to cement members of this phylum as widespread and diverse parasites infecting a broad range of eukaryotic microbes.

## Author summary

Little is known about the biology of bacteria in the candidate phylum Dependentiae, despite being widespread in nature. Here, we describe a novel isolate of this phylum, *Chromulinavorax destructans*, which infects an abundant aquatic predatory protist, *Spumella elongata*. *Chromulinavorax destructans* is an obligate intracellular parasite, forgoes binary fission and replicates surrounded by the host mitochondrion. The genome of *C*. *destructans* encodes no detectable complete metabolic pathways and instead contains extensive transporter systems to import metabolites and even energy in the form of ATP from the host. We also found a surprising number of genes in the *C*. *destructans* genome encoding putative host modifying proteins that might be responsible for the extensive host reorganization. Phylogenetic analysis showed that *C*. *destructans* is distantly related to intracellular pathogens and symbionts of unrelated amoebae. Hence, *Chromulinavorax destructans* provides new insights into the biology of a widespread but largely unknown phylum of bacteria. These results imply that members of the Dependentiae are pathogens of diverse aquatic protists and are therefore likely important players in aquatic ecosystems.

## Introduction

The candidate phylum Dependentiae (also known as TM6) is a phylogenetic group of bacteria that is only known from metagenomic data and from two isolates that infect free-living amoebae. Although relatively little is known about members of the Dependentiae, analysis of environmental 16S-ribosomal RNA sequences and metagenomic data indicate that they are widespread across diverse environments including peat bogs [1], hospital biofilms, soil, and waste water [2, 3]. Moreover, analysis of these data imply that bacteria in this phylum have very limited metabolic capability, but encode an extensive system of transporters, including ATP transporters, as well as genes functions enriched in bacterial endosymbionts such as *Chlamydia*, *Wolbachia*, and *Rickettsia*, suggesting that bacteria in the Dependentiae are symbionts or parasites that rely extensively on eukaryotic hosts for energy and metabolites [2, 3].

The only two previously isolated representatives of the Dependentiae were from amoebae, which are competent hosts to a vast array of bacterial, eukaryotic, and viral symbionts and pathogens [4, 5]. The first isolate, *Babela massiliensis*, is an obligate pathogen that causes lysis of *Acanthamoeba castellanii*, while *Vermiphilus pyriformis* maintains a stable relationship with its host, *Vermamoeba vermiformis*, indicating that life-history strategies differ among bacteria in the Dependentiae [4, 5]. Both isolates show unusual replication strategies within their hosts that delay cell fission and initially produce large replication bodies before dividing into progeny cells. Since the genome of *Babela massiliensis* is the only complete genome available for the members of the Dependentiae, a detailed comparison of the genomic complement involved in the different lifestyles is not possible; yet, several almost-complete metagenomically assembled genomes (MAGs) for other members of the phylum suggest that they are distinct from those infecting amoebae [6–8].

Nanoflagellate zooplankton, defined as being between 2 and 20 μm in size are major predators in aquatic systems, preying on bacteria, viruses, and other microbial eukaryotes [9, 10]. They are major engines of microbial mortality that link microbial biomass to higher trophic levels, and fuel biogeochemical cycling [11]. Within this broad functional group are many chrysophytes, a diverse group of nanoflagellates within the stramenopiles that includes phototrophs, mixotrophs and heterotrophs. A particularly widespread and abundant heterotrophic members of this group are members of the genus *Spumella* [12, 13]. These slightly elongated heterokont flagellates can also form amoeboid cells, occur in fresh and salt waters, and are readily isolated from many environments [14]. Little is known about the agents of mortality affecting members from this ubiquitous and ecologically important genus.

Here we present details on a representative isolate from the candidate phylum Dependentiae, *Chromulinavorax destructans*, which infects and lyses *Spumella elongata*. Its unusual replication cycle superficially resembles the infection cycle of a virus, and includes the creation of a replication ‘factory’ and the remodelling of the host mitochondrion. The genome is highly reduced and lacks nearly all genes related to energy metabolism, it encodes a large suite of transporters, including ATP transporters that illustrates its dependency on the host. Overall, *C*. *destructans* provides a link between molecular survey data and biological observations, and expands the known functional diversity and ecological roles of TM6 bacteria.

## Results

### *Chromulinavorax destructans* is a lytic pathogen of Spumella elongata

In an effort to isolate pathogens, such as giant viruses and bacteria, that infect ecologically relevant protistan zooplankton, microbial assemblages smaller than 0.8 μm from a variety of freshwater habitats in southwestern British Columbia were concentrated by filtration. These concentrates were pooled and used to inoculate cultures of protists isolated from the same environments, as well as from culture collections; cell densities were monitored by flow cytometry. One such screen yielded a pathogen infecting *Spumella elongata* (CCAP 955/1) that caused lysis of the culture within 48 h post infection (hpi). The lytic agent was named *Candidatus Chromulinavorax destructans* (after *Chromulinaceae*, the family of its host, “*vorax”*, meaning “gluttonous”, and “destructans”, meaning “destroying”; subsequently referred to as *C*. *destructans*), strain SeV-1. Infection was strain-specific and a different isolate of *Spumella sp*. (strain MK, 18S GenBank accession: MK782047) from one of the environments sampled for pathogen concentration did not cause lysis. The pathogen continued to cause lysis of *S*. *elongata* after storage at 4°C for up to four years.

Under epifluorescence microscopy, infected cells developed large DNA-containing intracytoplasmic compartments starting at 8 hpi, followed by lysis starting at 18 hpi that was associated with numerous DNA-containing particles of approximately 0.4 μm bursting out of the cytoplasm (S1B Fig). Flow cytometry revealed “free” *Chromulinavorax destructans* cells in the medium 12 hpi (S2B Fig), coinciding with a decline in host-cell density (S2A Fig). The population of *C*. *destructans* showed a homogeneous fluorescence signature and side-scatter heterogeneity suggests that the cells vary in size (S2B Fig).

**Fig 1:**
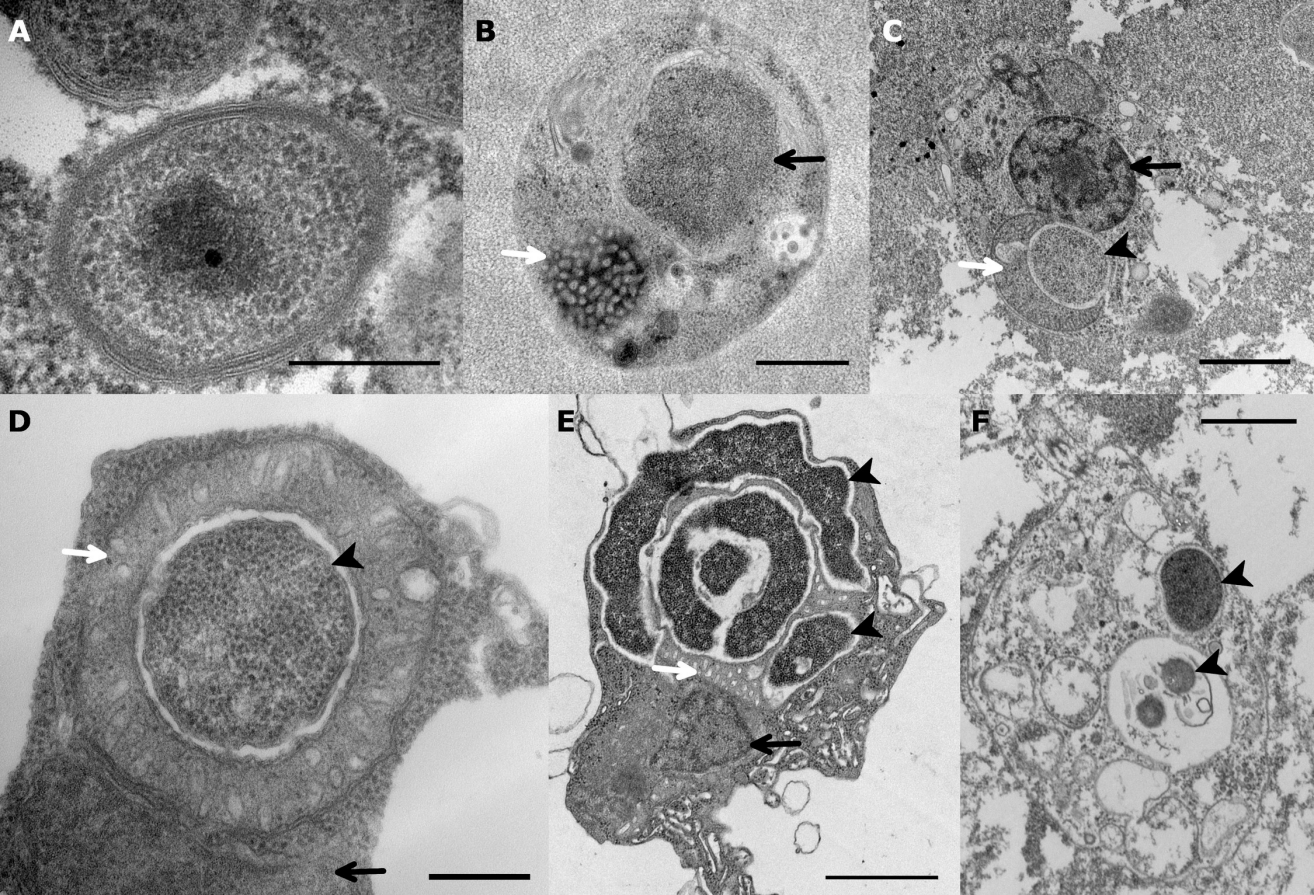
Micrographs of Chromulinavorax destructans. A: Thin-sectioned, high-pressure frozen electron micrographs of *C*. *destructans* reveal 350- to 400-nm particles with inner and outer membranes, as well as dark-staining periplasmic material. The genome is condensed into a nucleoid in the free particle. B: Electron micrograph of a healthy *Spumella elongata* cell showing the nucleus (black arrow) and the mitochondrion (white arrow). C: At 3 hpi, *C*. *destructans* (black arrow head) has invaded the host cytoplasm and the mitochondrion (white arrow) has wrapped around the replication body, while the nucleus stays intact (black arrow). D: Close-up of the replication body (black arrow head) surrounded by the host mitochondrion (white arrow) showing intact membranes of both the pathogen and the organelle (black arrow: nucleus). E: At 12 hpi, *C*. *destructans* shows long filaments and the beginning of septation (black arrowhead), surrounded by the highly invaginated mitochondrion (white arrow). The nucleus is present, but shows signs of degradation. F: Ghost cell of *S*. *elongata* with degraded cytoplasm and *C*. *destructans* (black arrow heads) at 18 hpi. Scale Bar: A,D: 250 nm; B: 500 nm; C,E,G: 1 μm.

In negative-staining electron microscopy, *C*. *destructans* cells present as 350–400 nm cocci with one polymorphous depression, similar to *Babela massiliensis* (S1C Fig) [5]. In thin-section electron microscopy, free *C*. *destructans* cells show a gram-negative-like coccoid morphology with a lipid double layer and electron-dense material in the periplasm (Fig 1A). The central part of the cytoplasm shows an electron-dense nucleoid. No depression similar to that seen in negative staining was observed in thin sections, suggesting that this depression is a preparation artefact of negative staining.

### *Chromulinavorax destructans* replicates in bodies nested in large mitochondrial invaginations

**Fig 2:**
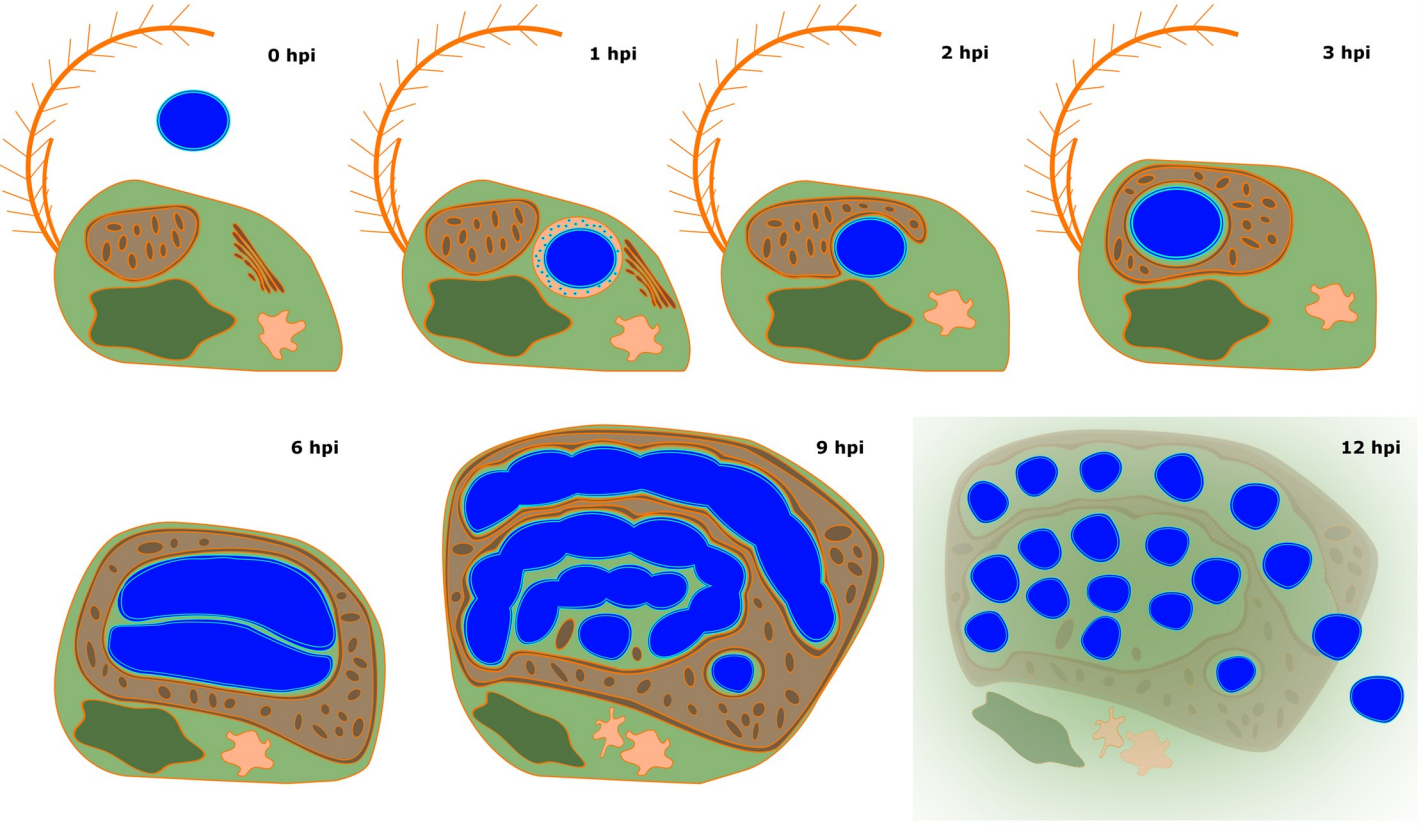
Schematic representation of the inferred replication cycle of Chromulinavorax destructans. The host S. elongata cell is shown in green, with the mitochondrion in brown, nucleus in dark green, Golgi in orange, and vacuoles in light orange. C. destructans is depicted in blue throughout its replication cycle.

The life cycle of the lytic bacterium *Chromulinavorax destructans* putatively begins when ingested by the phagotrophic protist, *Spumella elongata*, a heterokont and slightly elongated flagellate (Fig 1B, S1A Fig, Fig 2). Once in the food vacuoles, the cells appear to secrete outer membrane vesicles (S1E and S1F Fig), and by 3 hpi they appear as a spherical mass in the host’s cytoplasm with the mitochondrion partially wrapped around the bacterial replication body by forming a deep invagination (Fig 1C, Fig 2). The invagination becomes more pronounced over time until the mitochondrion completely surrounds the replication body. During this process, the membrane integrity of the parasitoid and the mitochondrion, including its cristae, remain intact, suggesting that there is no invasion of the mitochondrial matrix (Fig 1D, Fig 2). At 9 hpi, the bacterial replication bodies are surrounded by the mitochondrion, and appear as several amorphous elongated bodies (S1D Fig, Fig 2). This expansion phase culminates around 12 hpi, when the mitochondrion, now containing numerous invaginations and inclusions occupied by the replication bodies, takes up two thirds of the host cytoplasm (Fig 1E, Fig 2). Despite this extensive modification, the mitochondrion is still intact as membrane integrity is not compromised and cristae structures are preserved (Fig 1E, Fig 2). This integrity is contrasted by the degradation of the nucleus, as well as extensive membrane disarray inside the cell, including membrane vesicles budding from the cell (Fig 1E, Fig 2). The *C*. *destructans* replication bodies now show signs of regularly spaced invaginations, preceding division into the mature cocci (Fig 1E, Fig 2). The replication cycle completes at around 19 hpi, when *Spumella elongata* ghost cells with emptied cytoplasm appear (Fig 1F, Fig 2). The mature coccoid form of *C*. *destructans* are seen both intracellularly and extracellularly. Occasionally, replication bodies that did not complete the replication cycle, possibly due to prematurely exhausting the host cell’s resources, were observed within ghost cells (Fig 1F).

### The *Chromulinavorax destructans* genome encodes a minimal set of core functions but an extensive assortment of genes involved in host modification

**Fig 3:**
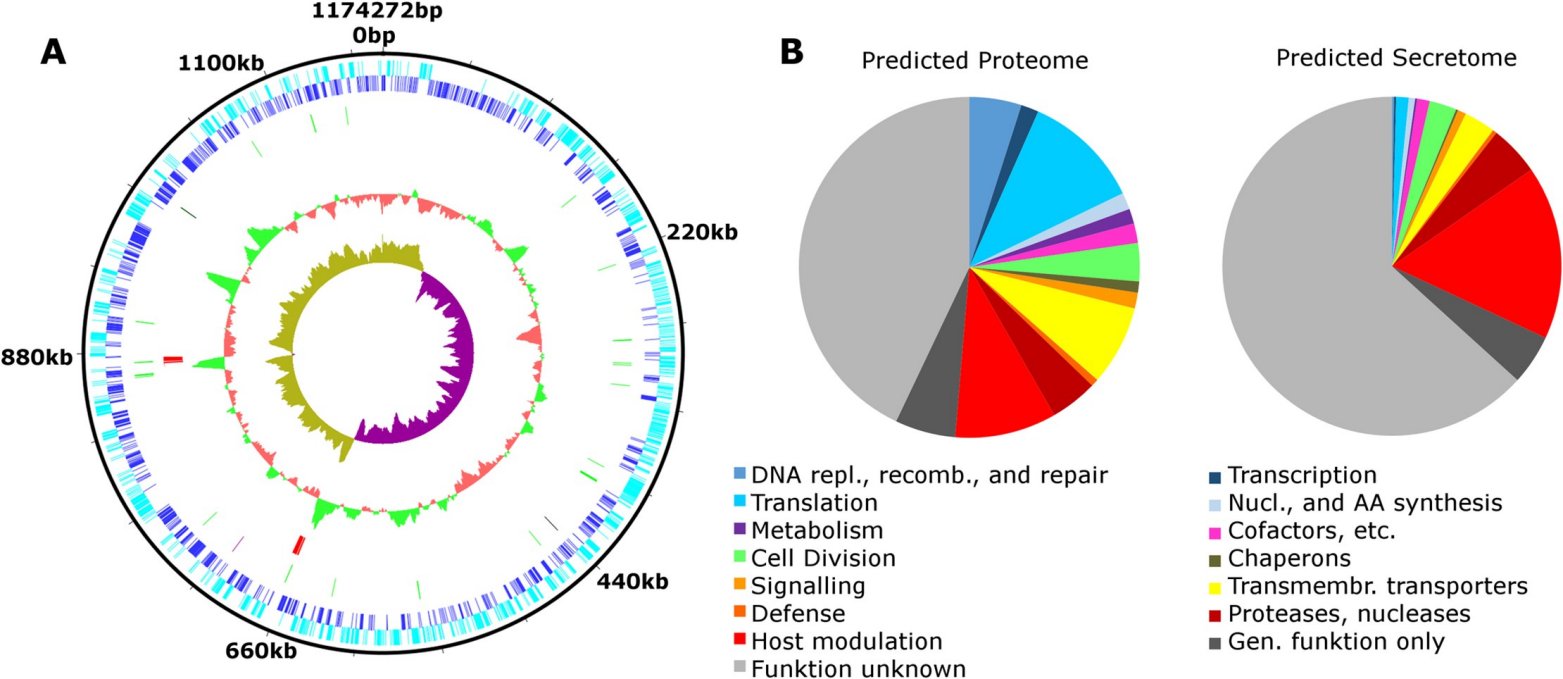
Genome content of Chromulinavorax destructans. A: Genome map showing open reading frames encoded by the plus and negative strand (dark and light blue respectively), tRNAs (green), rRNAs (red) and other ncRNAs (black). Inner circles depict GC content (red/green) and GC skew (teal/purple). B: Functional groups of putative protein-coding genes (Predicted Proteome) and the fraction coding a signal sequence (Predicted Secretome).

The 1,174,272 bp circular ds DNA *Chromulinavorax destructans* genome has a GC content of 33.7%, similar to other intracellular parasitoids of eukaryotes [5, 15]. GC skew and the location of the presumptive DnaA box “TTATCCACA“suggest that the origin of replication lies at 651641-652233bp (Fig 3A). The genome encodes two typical complete rDNA loci and 35 tRNAs covering all twenty amino acids (Fig 3A). Other ncRNAs include 4.8S RNA (*ffs* ncRNA), RNase P RNA (*rnpB* ncRNA), as well as a tmRNA (*ssrA*) (Fig 3A). Of the 1,081 predicted open reading frames 55% had a functional prediction with the largest fractions involved in DNA replication, translation, trans-membrane transporters and host manipulation (Fig 3B).

The DNA replication and repair machinery of *C*. *destructans* consists of a simple origin binding complex (lacking *dnaC*) and a typical prokaryotic replicase complex. DNA repair pathways include nucleotide excision repair (uvr system), short- and long-patch base-excision repair and mismatch repair (*mutL/S/Y* and *recJ*). Homologous recombination is supported by the *rec* and *ruv* pathways (with dpo), as well as site-specific recombinases (*xerJ/D*). The RNA polymerases assisted in transcription by several transcriptional regulators (*merR*, *yebC*, *fmdB*), transcription termination factors (*nusA/B/G*), and RNase H.

The ribosome, missing proteins 30S-22, 50S-8, 50S-25, 50S-26, 50S-30 and the rRNAs are edited by several ribosomal modification enzymes such as methyl and acetyl transferases. Similarly, the tRNAs are modified by a large number of modifying enzymes such as methyl transferases and dihydrouridine synthases and are loaded by a full set of 21 aminoacyl-tRNA synthetases. Peptide chain release factors (*prfA/B*), ribonucleases (RNaseY/P), ribosome recycling factor (*rrf*), translation initiation and elongation factors promote translation (*infA/B/C*, *fusA*, *lepA*, *elf*, *tsf*, *tufB*). Post translational modifications are assisted by several chaperones and some proteins are subjected to transport to membranes.

*Chromulinavorax destructans* utilizes nucleoside salvage for both purine and pyrimidine biosynthesis and is able to phosphorylate the products into all required nucleotides. Therefore, precursor nucleosides must be imported from the host. Similarly, amino-acid biosynthesis is restricted to simple conversions between related amino acids (ser/cys, cys/ala, glu/gln, ser/gly), emphasizing that *C*. *destructans* is highly dependent on the host.

*Chromulinavorax destructans* encodes very rudimentary cell-division machinery, with only *ftsA*, *K*, *L*, *W*, and *Z* present, supported by *zapA*. Cell shape is determined by *mreB*, *C*, and *RodA*. Despite the microscopic observations of a gram-negative-like phenotype, no LPS biosynthesis genes were observed. However, a complete *mur* pathway of peptidoglycan biosynthesis is likely responsible for generating the electron-dense material in the periplasm observed in electron micrographs (Fig 1A). Several surface antigens of unknown function are also encoded, as well as Type IV pilus assembly proteins, which might be assembled through a derived *pulD* channel. Several signaling trans-membrane receptors and signal-transduction proteins such as *ispA*, *ftsY*, or *spoVR* could influencing the cell cycle are putatively involved, which could be involved in switching from the free inactive state to active replication.

As there was no evidence for complete metabolic pathways encoded by the genome of *C*. *destructans*, it implies that the cells must rely on extensive transmembrane transport systems to import metabolites and other resources from its host (S4 Fig). A large array of ABC transporters involved in importing oligopeptides and amino acids through the *pot* and *opp/dpp* systems, presumably provide amino acids for protein synthesis. Phospholipids and lipoproteins are imported through the *mal* and *lol* ABC transporter systems respectively. ABC transporter systems *fep* and *tro* import trace elements such as iron, zinc and manganese (*znu*). Other ABC transporter systems of uncharacterized specificity are present, including putative multidrug exporters. Besides the ABC transporter systems, several specialized symporters, antiporters, and pumps are predicted to import potassium (*trk*), sodium (*nha/als/put*), inorganic ions (*mgt*), and nucleosides (*nup*). Further, specialized multidrug exporters are also present (*rhat/eama*). Central to energy requirements, there are two copies of a *tlcc* ATP/ADP antiporter that allows for the exchange of ADP for ATP from the host, which seems to be the only source of ATP. Membrane potential is essential for the function of many transporters and antiporters. This potential appears to be maintained by an ATP-synthase running in reverse, exporting protons in an ATP dependent manner, which may be regulated by passive proton channels. Large biomolecules can be imported by mechanosensitive channels (*msc*) and a biopolymer transporter (*exb*).

A substantial proportion of the genome encodes proteases, nucleases, and hydrolases, which are putatively involved in processing and breaking down host-cell structures and biopolymers that can be imported and reused. These effectors can be classified as secreted, membrane bound, and cytoplasmic factors. Amongst the secreted hydrolases is a close relative of chitinases and several HAD superfamily hydrolases (*yigB*, *mphC*, *glcD*). Secreted proteinases include M3, M15, M23, M41, M48, C11, and C65 peptidases. C65, M23, and M50 peptidases are predicted to be membrane bound. The final step in host biopolymer degradation is accomplished by cytoplasmic endonucleases (*endoU*), hydrolases (NUDIX and HIT-type), and proteases (lon, M16, M17). As well, a putative superoxide dismutase is encoded, which could help the cell cope with stressors such as hydrogen peroxide from close proximity to the mitochondrion.

More than 10% of the proteins encoded by *C*. *destructans* are predicted to be involved in modifying and influencing the host cell. Most prominent are 98 copies of ankyrin-repeat domain proteins. This concurs with observations of other intracellular parasitoids among the TM6 candidate phylum, such as *Babela massiliensis*, but also includes unrelated intracellular parasites such as *Legionella* spp., and giant viruses [5, 16–18]. The exact function of ankyrin-repeat domain proteins is unknown, but they have been implicated in membrane modification and counteracting the host immune system [17, 19, 20]. A CDS for a protein distantly resembling mitofilin was also found and is an intriguing candidate for causing the extensive manipulation of the mitochondrion.

Similar to genes thought to engage in host interaction, there are several sequences that putatively code for proteins involved in interacting with competing symbionts or pathogens, including antibiotic resistance factors such as a Bacitracin-resistance protein or the L-ascorbate metabolism protein UlaG; however, complete pathways were not evident. In addition, quorum-sensing mechanisms as well as CRISPR loci were absent.

A *sec* secretion system is likely used to export proteins in the periplasm and to secrete proteins. Interestingly, 44% of all CDS possess a putative signal peptide that targets them to the secretion system, either as membrane proteins or as secreted proteins. This subset of secreted proteins is enriched in ORFans and proteins of unknown function compared to the complete set of CDS (Fig 3B). However, other overrepresented fractions include putative CDS for proteins that break down or alter the host, such as proteinases and nucleases, and most prominently the ankyrin-repeat domain proteins, 78% of which contain a signal protein.

### Phylogenetic position of *Chromulinavorax destructans* within the candidate phylum Dependentiae

**Fig 4:**
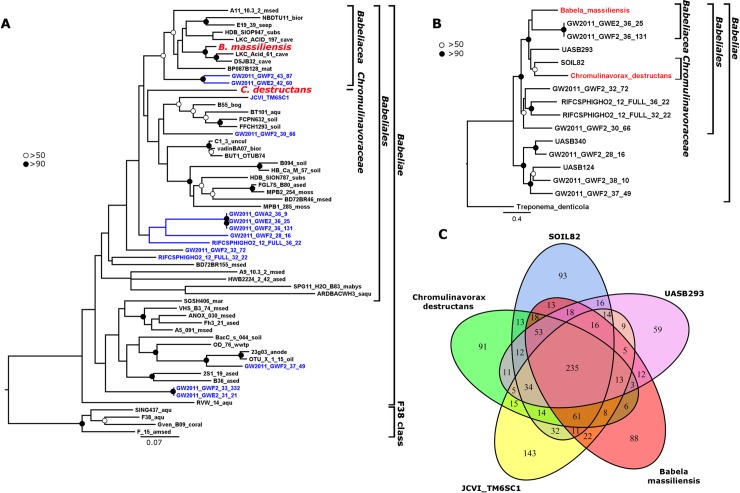
Phylogenetic placement of Chromulinavorax destructans. A) 16S full length rDNA ML tree (1000 BS replicates) showing the Dependentiae phylum. “Substantially complete” MAGs highlighted in blue, isolates in red. Solid circles show node support of >90 and open circle >50. B) ML tree of ribosomal proteins (L2, L3, L4, L5, L6, L14, L15, L16, L18, L22, L24, S3, S8, S10, S17, S19; BS support based on 1000BS replicates). C) Shared gene clusters between *C*. *destructans* and members of its family *Chromulinavoraceae* (UASB293, SOIL82, and JCVI TM6SCI) and *Babela massiliensis* as a representative of the *Babeliaceae*.

Full length 16S rDNA maximum likelihood (ML) phylogenetic analysis of the Dependentiae places *C*. *destructans* into the proposed order *Babeliales* and within the class *Babeliae* [3], but as a sister family to the *Babeliaceae*, for which the name *Chromulinavoraceae* (fam. nov.) is proposed (Fig 4A). The proposed family is separate from the clade containing the original MAG of JCVI_TM6SC1 and, based on a partial 16S sequence, also separate from *Vermiphilus pyriformis* and its proposed family *Vermiphilaceae* [4]. The basal nodes in the 16S rDNA ML tree are poorly supported, likely due to undersampling, and similar to a previous analysis [3].

The phylogenetic position of *C*. *destructans* was also explored by ML analysis of concatenated ribosomal proteins from MAGs that confidently contained all of the ribosomal CDSs investigated (Fig 4B). The resulting tree reflects the architecture of the 16S rDNA tree and supports the proposed *Chromulinavoraceae* as a sister family to the *Babeliaceae* within the order *Babeliales*, which is distinct from a second well-supported order of environmental sequences (Fig 4B). Genome-content analysis of representatives of the two families within the *Babeliales* supports the separation of the families *Babeliaceae* and *Chromulinavoraceae*. The proposed members of the *Chromulinavoraceae* share more clusters of orthologous genes with each other than with *Babela masiliensis* of the *Babeliaceae*, despite the *Chromulinavoraceae* including a potentially incomplete MAG (SOIL82) and the *Babeliaceae* being represented by complete genomes (Fig 4C).

Similarly, full genome taxonomical analysis against the Genome Taxonomy Database clustered *C*. *destructans* confidently within the order *Babeliales*, while occupying a separate family from the *Babeliaceae* and the recently proposed *Vermaphilaceae* (S3 Fig). One previous MAG (GB_GCA_002721975.1) in the Genome Taxonomy Database that was assigned to the *Chromulinavoraceae* originated form a pelagic metagenomic sample from the South Pacific Ocean; it showed a relative evolutionary divergence of 0.789 suggesting divergence at genus level [21, 22].

## Discussion

### *Chromulinavorax destructans* is highly host-dependent

**Fig 5:**
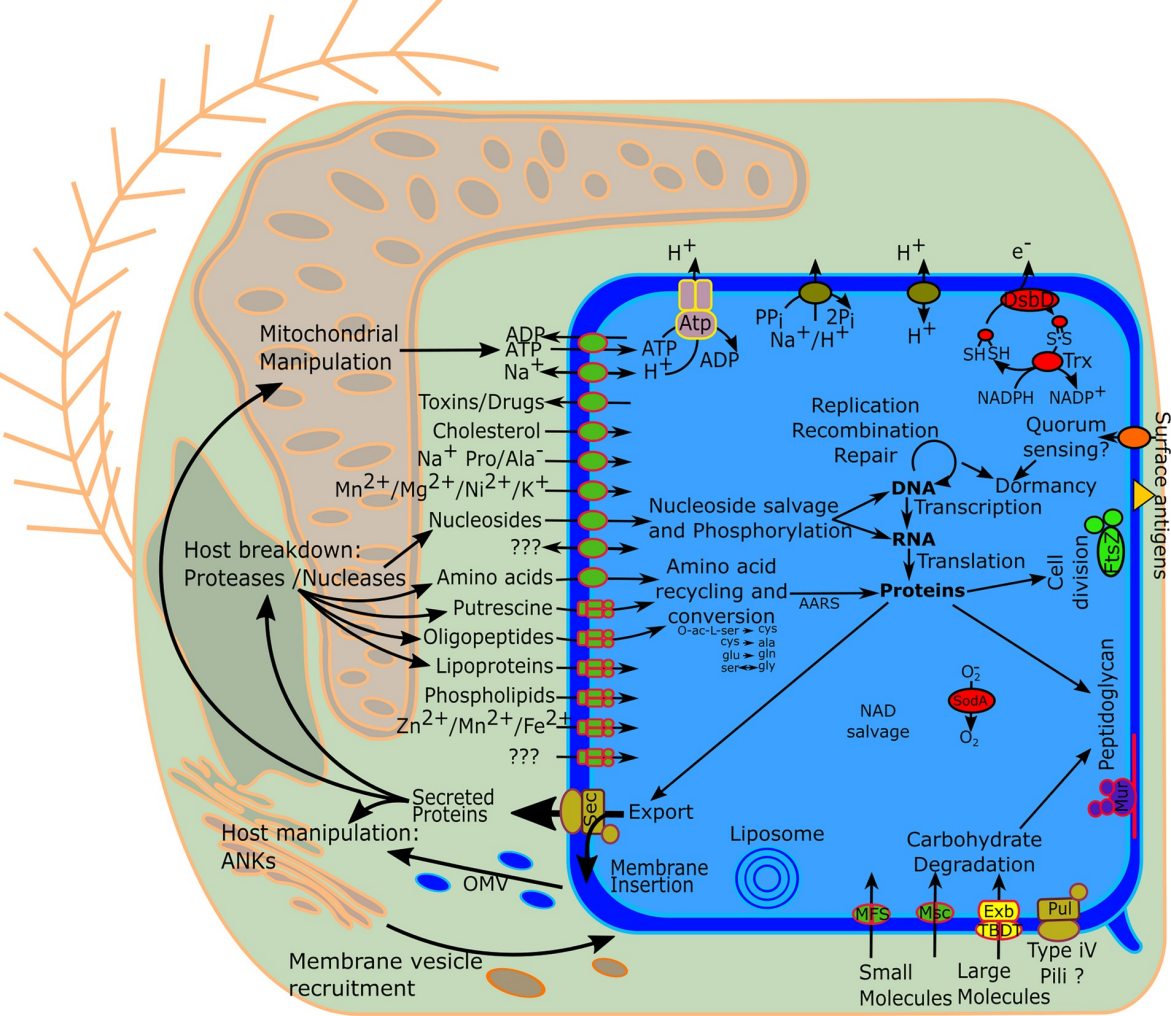
Schematic representation of inferred metabolic capabilities of Chromulinavorax destructans and its interactions with its host, Spumella elongata.

*Chromulinavorax destructans* is an intracellular pathogen of *S*. *elongata* that appears to be dependent on its host for replication, to the extent that it does not appear to encode any complete metabolic pathways (Fig 5, S4 Fig). Many putative effector molecules, such as proteases, nucleases and ankyrin–repeat domain proteins, that could manipulate and break down host structures contain signal peptides and appear to be secreted from the bacteria (Fig 3). Similarly, putative outer membrane vesicles that could also contain such effector molecules bud during the early stages of infection and might serve a similar function (S1E and S1F Fig). The reorganization and breakdown of host structures provide resources, possibly due to the aforementioned effector molecules, that can be imported into the C. *destructans* replication bodies (Fig 5). With no evidence of lipid biosynthesis, in combination with the observed host membrane disarray during late infection, suggests that host lipids are used for the bacterial cell membrane. Remodeling of the host mitochondrion is one of the most unusual features of the replication process. The expansion of the mitochondrion while maintaining membrane integrity contrasts with observations from other mitochondrion-invading pathogens and symbionts that actively disrupt the mitochondrial integrity [23, 24]. The proximity of bacterial replication to the expanded mitochondrion would allow for a steady supply of ATP mediated by the encoded ADP/ATP antiporter, but it would also require free radicals to be neutralized, a function likely performed by the bacterial encoded superoxide dismutase, also found in other members of the Dependentiae [2, 3, 5]. This high host-dependence suggests the pathogen is inactive until taken up by a host cell,which may also be the reason that the cells remained infectious after four years at 4°C in the absence of host cells. Complete host dependence and extracellular inactivity are life-cycle characteristics that highlight traits of convergent evolution found in many obligate pathogens. For example, Microsporidia completely lack energy generation pathways, while ATP transporters are widespread [25–27]. The similarity in life style and genome content, such as dedicating more than then percent of their genome to ankyrin-repeat domain proteins, between Dependentiae and giant viruses provides an intriguing example of convergent evolutionary trajectories of obligate pathogens from vastly different evolutionary backgrounds [5, 16].

### The extensive mitochondrial modifications caused by *Chromulinavorax destructans* might stem from limited host resources

A rudimentary cell-division machinery and the lack of many hallmark genes (e.g. *ftsN*, *zapB*, and *zipA*) has been highlighted for members of the Dependentiae based on MAGs [2, 3]. Similarly, *Babela massiliensis* showed delayed cell division after initial growth in amorphous bodies (i.e. replication bodies), and, while not directly described, *Vermiphilus pyriformis* likely uses a similar replication strategy [4, 5]. Although the replication of *C*. *destructans* in replication bodies resembles *B*. *massiliensis*, the close association and modulation of the mitochondrion was unique to *C*. *destructans*, possibly partially mediated by the mitofilin-like protein encoded exclusively by *C*. *destructans* among the Dependentiae. In part, this difference might reflect the availability of cytoplasmic resources, such as metabolites and ATP, that presumably are scarcer in the flagellate cell compared to the much larger amoebae that are hosts to the two previously described isolates; Accordingly, *C*. *destructans* causes severe deformation of the cellular architecture of the nanoflagellate that was not seen in amoebae hosts (Figs 1 and 2, S1 Fig). The scarcity of cytoplasmic ATP could lead to the close association of *C*. *destructans* with the host mitochondrion and initiate the mitochondrion’s expansion and rearrangement. A limitation of energy could have led to the incomplete replication bodies that were frequently found inside ghost cells, suggesting that ATP availability might restrain *C*. *destructans* replication.

The differences between amoeba-infecting and nanoflagellate-infecting Dependentiae resemble observations made for giant viruses infecting different hosts. Giant viruses infecting nanoflagellates show highly spatially oriented replication and preserve the mitochondrion; whereas, amoeba-infecting viruses seem to be unrestricted by the host-cell architecture [16, 28]. Additionally, a similarly high percentage of the genome of the lytic Dependentiae representatives is dedicated to ankyrin-repeat domain proteins, suggesting that these are crucial effectors for intra-eukaryotic replication and host cell take-over [5, 16]. Although the specific function of these proteins within these host-pathogen systems remain unknown, their nature as protein-protein interaction domains suggests they might be involved in host modification strategies [17]. Where ankyrin-repeat domain proteins have been explored in other host-pathogen systems, their functions include blocking of the host immune system, the induction of phagocytosis, the facilitation of cytoplasmic invasion, and the self-protection from potentially damaging effector molecules [17, 20, 29–31]. Any one of these functions could benefit *C*. *destructans* during its replication and therefore might be deployed.

### Life history strategies and host-range might differ vastly amongst the Dependentiae and might promote diversity within the phylum

Phylogenetic analysis confidently places *C*. *destructans* within the candidate phylum TM6/Dependentiae, making it the first reported isolate of the phylum that does not infect an amoeba, and the first to infect a representative of the stramenopile supergroup of eukaryotes (Fig 4). Other studies have reported metagenomic correlation of Dependentiae with ciliates, suggesting that they infect or are symbionts of other eukaryotic lineages [3]. Life-history strategy seems to be independent of the host taxonomic group, given that *C*. *destructans* and *B*. *massiliensis* are lytic, but infect distantly related hosts, while *V*. *pyriformis* is symbiotic, and like *B*. *massiliensis* infects an amoeba. This suggests that life-history strategy, and resulting pathogenicity, is a product of coevolution within a specific host-pathogen pair. The restricted host ranges of *C*. *destructans* and *V*. *pyriformis* are consistent with members of the Dependentiae being highly adapted to their hosts [4]. The highly restricted host range of members of the Dependentiae suggests that they are extraordinarily diverse, as is reflected by the great diversity of related 16S rRNA sequences from environmental surveys. Given their potentially broad host range and distribution, Dependentiae could play a significant role in controlling the abundance and diversity of aquatic autotrophic and heterotrophic protists.

### Summary

*Chromulinavorax destructans* is the first described isolate of the candidate phylum Dependentiae that does not infect *Acanthamoeba* and is the first isolate of the proposed family *Chromulinavoraceae* that has only been represented by MAGs. Replication causes extensive reorganization of the host cell, most notably the wrapping of the host mitochondrion around the replication bodies of the bacteria. The genomic complement of *C*. *destructans* is highly reduced in metabolic capabilities, with no evidence for encoding any complete metabolic pathway and, thus, relies extensively on host resources such as metabolites and even ATP for energy supply. Strikingly, *C*. *destructans* is rich in putative effector molecules, putatively breaking down and reorganizing its host, as well as encoding a large number of uncharacterized proteins and proteins not represented in databases. The narrow host range of *C*. *destructans* provides hints to a regulatory role of parasitic bacteria in the diversity and abundance of heterotrophic protists.

## Materials and methods

### Ethics statement

Sampling of aquatic environments was approved by the University of British Columbia.

### Sampling

Samples were collected from 11 freshwater locations in southern British Columbia, Canada (49°49'4"N, 123° 7'46"W; 49°42'5"N, 123° 8'47"W; 49°37'34"N, 123°12'27"W; 49° 6'12"N, 122° 4'38"W; 49° 5'22"N, 122° 7'1"W; 49°18'10"N, 122°42'9"W; 49° 8'27"N; 123° 3'16"W; 49°13'21"N, 123°12'43"W; 49°13'13"N, 123°12'41"W; 49°14'52"N, 123°13'59"W; 49°15'58"N, 123°15'34"W). To concentrate pathogens, 20 liter water samples were prefiltered with a GF-A filter (Millipore, Bedford, MA, USA; nominal pore size 1.1 μm) over a 0.8- μm pore-size PES membrane (Sterlitech, Kent, WA, USA) [32]. Filtrates from all locations were pooled and concentrated using a 30kDa MW cut-off tangential flow ultrafiltration cartridge (Millipore, Bedford, MA, USA) [33].

### Isolation

The host organism, *Spumella elongata* strain CCAP 955/1 was kindly provided by David Caron (University of Southern California) and maintained in modified DY-V artificial fresh water media with yeast extract and a wheat grain [34]. *Spumella elongata* strain CCAP 955/1 was one of 49 protist cultures from diverse phylogenetic backgrounds that were used in a larger screening effort to isolate parasites of heterotrophic protists (for more details on the screen, see Deeg et. al 2018 [16]). Cultures of *S*. *elongata* at approximately 2x10^5^ cells/ml were inoculated with the pooled microbial concentrates from all 11 locations. Cell numbers of the inoculated culture were monitored by flow cytometry and compared to a medium-only mock-infected control culture using flow cytometry (LysoTracker Green (Molecular Probes) vs. FSC on FACScalibur (Becton-Dickinson, Franklin Lakes, New Jersey, USA)) [35]. After cell lysis, the lysate was filtered through a 0.8-μm pore-size PES membrane (Sterlitech) to remove remaining host cells. The lytic agent was propagated and made clonal by three serial end-point dilutions. The concentrations of the lytic agent were screened by flow cytometry using SYBR Green (Invitrogen Carlsbad, California, USA) nucleic-acid stain after 2% glutaraldehyde fixation (vs SSC). The flow cytometry profile presented as a population clearly distinct from heterotrophic bacteria, phage, and eukaryotes; however, their larger size heterogeneity when compared to giant virus isolates suggested a non-viral nature [16].

### Transmission electron microscopy

#### Negative staining

Lysates of *Spumella elongata* after infection with *Chromulinavorax destructans* were applied to the carbon side of a formvar carbon-coated 400-mesh copper grids (TedPella, CA, USA) and incubated at 4°C in the dark overnight in the presence of high humidity. Next, the lysate was removed and the grids were stained with 1% Uranyl acetate for 30 s.

#### Ultra-thin sectioning

Exponentially growing cultures of S. elongata at a concentration of 5x105 cells ml-1 were infected with C. destructans at a ratio of ~5 pathogen to host cells to ensure synchronous infection. Cells were harvested from infected cultures at 3, 6, 9, 12, 18, and 24 h post infection, as well as from uninfected control cultures. Cells from 50 ml were pelleted in two consecutive 10 min at 5000 xg centrifugation runs in a fixed angle Beckmann tabletop centrifuge.

For chemical fixation, the pellet was resuspended in 0.2 M Na cacodylate buffer, 0.2 M sucrose, 5% EM grade glutaraldehyde, pH 7.4 and incubated for 2 h on ice. After washing in 0.2 M Na cacodylate buffer, cells were postfixed with 1% osmium tetroxide. Samples were dehydrated through water/ethanol gradients and ethanol was substituted by acetone. An equal part mixture of Spurr’s and Gembed resin was used to embed the cells, which was polymerized at 60°C overnight.

For high pressure freezing, cell pellets were resuspended in 10–15 μl of DY-V culture medium with 20% (w/v) BSA and immediately placed on ice. Cell suspensions were cryo-preserved using a Leica EM HPM100 high-pressure freezer. Vitrified samples were freeze-substituted in a Leica AFS system for 2 d at -85°C in a 0.5% glutaraldehyde / 0.1% tannic acid solution in acetone, then rinsed ten times in 100% acetone at -85°C, and transferred to 1% osmium tetroxide, 0.1% uranyl acetate in acetone and stored for an additional 2 d at -85°C. The samples were then warmed to -20°C for 10 h, held at -20°C for 6 h to facilitate osmication, and then warmed to 4°C for 12 h. The samples were then rinsed in 100% acetone 3X at room temperature and gradually infiltrated with an equal part mixture of Spurr’s and Gembed embedding media. Samples were polymerized in a 60°C oven overnight. Fifty nm thin sections were prepared using a Diatome ultra 45° knife (Diatome, Switzerland) on an ultra-microtome. The sections were collected on a 400x copper grid and stained for 10 min in 2% aqueous uranyl acetate and 5 min in Reynold’s lead citrate. Image data were recorded on a Hitachi H7600 transmission electron microscope at 80 kV. Image J (RRID:SCR_003070) was used to compile all TEM images. Adjustments to contrast and brightness levels were applied equally to all parts of the image.

### Pathogen concentration and sequencing

For PacBio sequencing, exponentially growing *S*. *elongata* cultures at a concentration of approximately 5x10^5^ cells ml^-1^ were infected with *C*. *destructans* lysate (~10^7^ cells ml^-1^) at a multiplicity of infection (MOI) of ~0.5. After four days, when host cells were undetectable, cultures were centrifuged in a Sorvall SLC-6000 centrifuge with fixed angle rotor for 20 min and 5000 rpm at 4°C to remove remaining host cells and the supernatant was subjected to tangential flow filtration with at 30kDa cut-off (Vivaflow PES) and concentrated approximately 100x. Concentrates were ultracentrifuged at 28,000 rpm, 15°C for 8h in a Ti90 fixed-angle rotor (Beckman-Coulter, Brea, California, USA). Next, the concentrate was further concentrated by sedimenting it onto a 40% Optiprep 50 mM Tris-Cl, pH 8.0, 2mM MgCl_2_ cushion for 30 min at 28,000 rpm and 15°C in a SW40Ti swinging-bucket rotor in an ultracentrifuge (Beckman-Coulter, Brea, California, USA). An Optiprep (Sigma) gradient was created by underlaying a 10% Optiprep solution in 50 mM Tris-Cl, pH 8.0, 2 mM MgCl_2_ with a 30% Optiprep solution followed by a 50% Optoprep solution and was equilibration overnight at 4°C. One ml of concentrate from the 40% cushion was added atop the gradient and the concentrate was fractionated by ultracentrifugation in an SW40 rotor for 4 h at 25000 rpm and 18°C. The fraction corresponding to the pathogen was extracted from the gradient with a syringe and washed twice with 50 mM Tris-Cl, pH 8.0, 2 mM MgCl_2_ followed by centrifugation in an SW40 rotor for 20 min at 7200 rpm and 18°C and finally collected by centrifugation in an SW40 rotor for 30 min at 7800 rpm and 18°C. Purity of the concentrate was verified by flow cytometry (SYBR Green (Invitrogen Carlsbad, California, USA) vs SSC on a FACScalibur flow cytometer (Becton-Dickinson, Franklin Lakes, New Jersey, USA). High molecular weight genomic DNA was extracted using phenol-chloroform-chloroform extraction. Length and purity of the DNA were confirmed by gel electrophoresis and a Bioanalyzer 2100 with the HS DNA kit (Agilent Technology). PacBio RSII 20kb sequencing was performed by the sequencing center of the University of Delaware. Reads were assembled using PacBio HGAP3 software with 20 kb seed reads and resulted in a single contig of 1,228,924bp, 819.19x coverage, 100% called bases and a consensus concordance of 99.9839% [36].

### Annotation

The genome was circularized, resulting in a final chromosome size of 1,174,272 bp. Genome annotation was performed using the automated NCBI Prokaryotic Genome Annotation Pipeline (PGAAP). In parallel, open reading frames were predicted using GLIMMER (RRID:SCR_011931) with default settings [37]. Translated proteins were analyzed using BLASTp, CDD RPS-BLAST and pfam HMMER. These results were used to refine the PGAAP annotation. Signal peptides and trans-membrane domains were predicted using Phobius [38]. The annotated genome is available under the accession number CP025544. Metabolic pathways were predicted using the Kyoto Encyclopedia of Genes and Genomes (KEGG RRID:SCR_012773) automatic annotation server KAAS and Pathway Tools (RRID:SCR_013786) [39, 40].

### Phylogenetic analysis

Full length 16S rDNA sequences belonging to the candidate phylum TM6 were downloaded from NCBI. Alignments of rDNA sequences were done in Geneious R9 (RRID:SCR_010519) using MUSCLE with default parameters (RRID:SCR_011812)[41]. Maximum likelihood trees were constructed with RAxML ML search with 1000 rapid bootstraps using GTR+GAMMA [42].

Near-complete metagenomically assembled genomes (MAGs) and complete genomes of isolates were retrieved from NCBI. There were 3820 clusters of orthologous genes defined by OrthoMCL (RRID:SCR_007839) with standard parameters (Blast E-value cut-off = 10^−5^ and mcl inflation factor = 1.5) on all protein coding genes of length ≥ 100 aa [43]. Overlap in gene content was defined using a custom R script (see Deeg et al. 2018 [16]). Genes encoding ribosomal proteins were identified by BLAST. Ribosomal protein L2, L3, L4, L5, L6, L14, L15, L18, L16, L22, L24, S3, S8, S10, S17, and S19 sequences were aligned using MUSCLE with default parameters (RRID:SCR_011812) [41]. Maximum-likelihood trees were constructed with RAxML rapid bootstrapping and ML search with 1000 bootstraps utilizing GTR+CAT [42].

Taxonomic classification according to the Genome Taxonomy Database was performed using GTDB-Tk (v0.2.2: https://github.com/Ecogenomics/GTDBTk) using the default classification workflow with the full *C*. *destructans* genome as the only query [22, 44–48].

## Supporting information

S1 Fig Micrographs of Chromulinavorax destructans. A: Epifluorescence micrograph of DAPI-stained *Spumella elongata* (prominently-stained nucleus). B: Epifluorescence micrograph of two *S*. *elongata* cells 19 h after exposure to *C*. *destructans*. Coccoid cells of *C*. *destructans* are seen bursting from a cell undergoing lysis. C: Purified *C*. *destructans* cell in negative-staining electron micrographs show a depression on the cell surface. D: Extensive *C*. *destructans* replication inside *S*. *elongata* at 9 hpi. E: Putative *C*. *destructans* (black arrow head) inside the food vacuole of *Spumella elongata* showing apparent secretion of outer membrane vesicles (white arrow head) and an intact S. elongata mitochondrion (white arrow). F: Putative *C*. *destructans* (black arrow head) inside the food vacuole of *Spumella elongata* showing liposomes and outer membrane vesicles (white arrow head). Scale bars: A,B: 5 μm, C,E,F: 250 nm, D: 500nm.
(TIF)

S2 Fig Effect of addition of Chromulinavorax destructans to a culture of Spumella elongata: A: Change in the cell number of S. elongate with (infected) and without (control) exposure to C. destructans added at day 0 post infection (background 8x104 cells ml-1). The error bars represent the SEM of triplicate cultures. B: Flow cytometric profile at 48hpi of C. destructans (red arrow, identified by purification and sequencing) in a culture of S. elongata and stained with SYBR-Green. Chromulinavorax destructans shows a homogeneous genome size (constant green fluorescence signal across the population). Other populations of bacteria with larger genomes (populations with higher green fluorescence levels) are present in the culture and show the typical sloped population indicative of genome replication (higher side scatter correlates with higher green fluorescence).
(TIF)

S3 Fig Phylogenetic placement of Chromulinavorax destructans. Maximum likelihood tree of the order Babeliales based on genome taxonomy toolkit analysis (Shimodaira-Hasegawa local support values shown; isolates highlighted in red). *Chromulinavorax destructans* represents a separate family from the *Babeliaceae* and the recently proposed *Vermaphilaceae* within this order. The only other genus in the family *Chromulinaovoraceae* is represented by an incomplete MAG from the South Pacific Ocean.
(TIF)

S4 Fig Metabolic pathways of Chromulinavorax destructans reconstructed by PathwayTools. (TIF)
