# Supplementary figures and images for: Chromulinavorax destructans, a pathogen of microzooplankton that provides a window into the enigmatic candidate phylum Dependentiae
Source: PLoS Pathog. 2019 May 31;15(5):e1007801. doi: 10.1371/journal.ppat.1007801 (PMC6561590; doi:10.1371/journal.ppat.1007801)

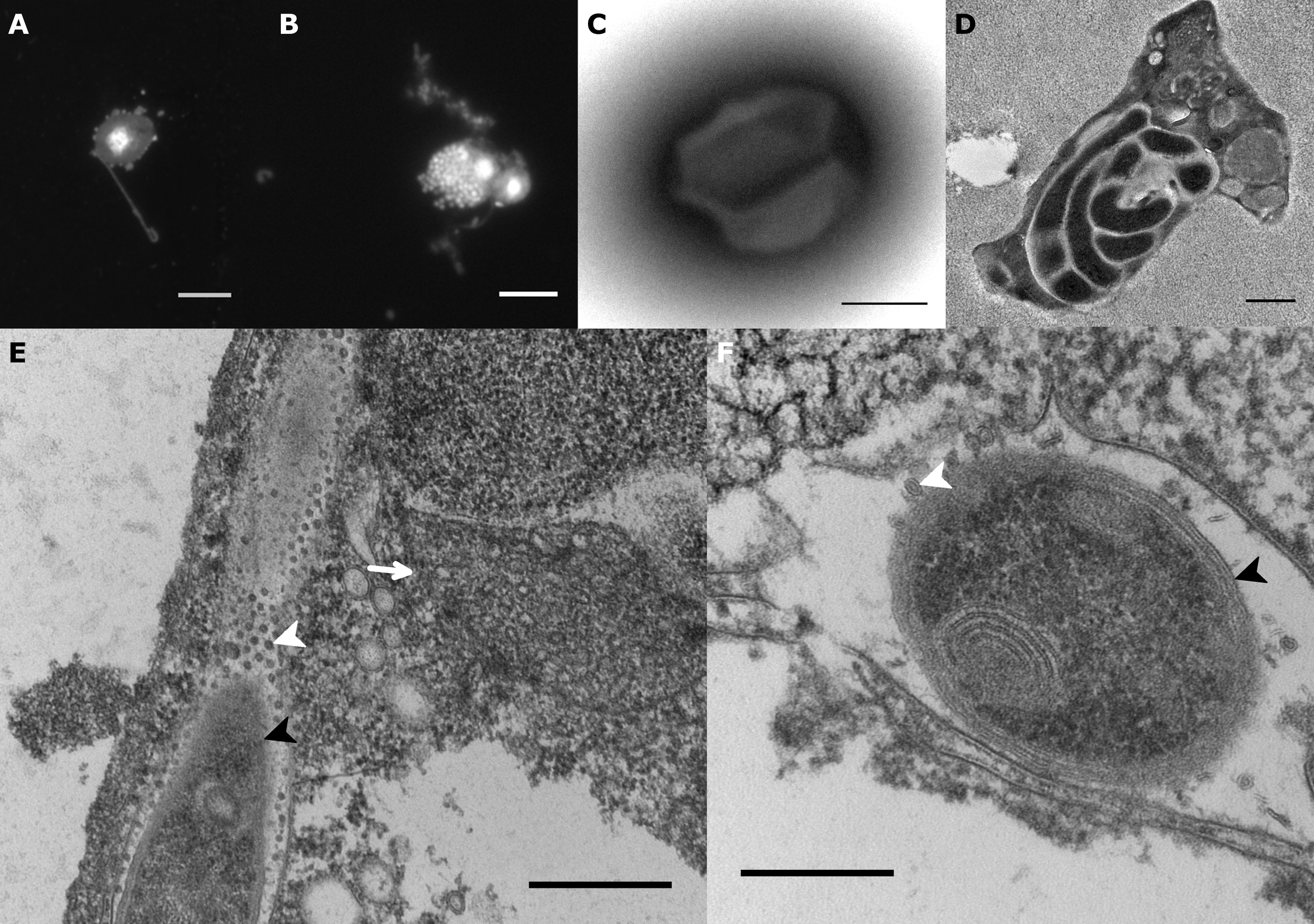

Supplement: S1 Fig — A: Epifluorescence micrograph of DAPI-stained Spumella elongata (prominently-stained nucleus). B: Epifluorescence micrograph of two S. elongata cells 19 h after exposure to C. destructans. Coccoid cells of C. destructans are seen bursting from a cell undergoing lysis. C: Purified C. destructans cell in negative-staining electron micrographs show a depression on the cell surface. D: Extensive C. destructans replication inside S. elongata at 9 hpi. E: Putative C. destructans (black arrow head) inside the food vacuole of Spumella elongata showing apparent secretion of outer membrane vesicles (white arrow head) and an intact S. elongata mitochondrion (white arrow). F: Putative C. destructans (black arrow head) inside the food vacuole of Spumella elongata showing liposomes and outer membrane vesicles (white arrow head). Scale bars: A,B: 5 μm, C,E,F: 250 nm, D: 500nm. (TIF) [file ppat.1007801.s001.tif]

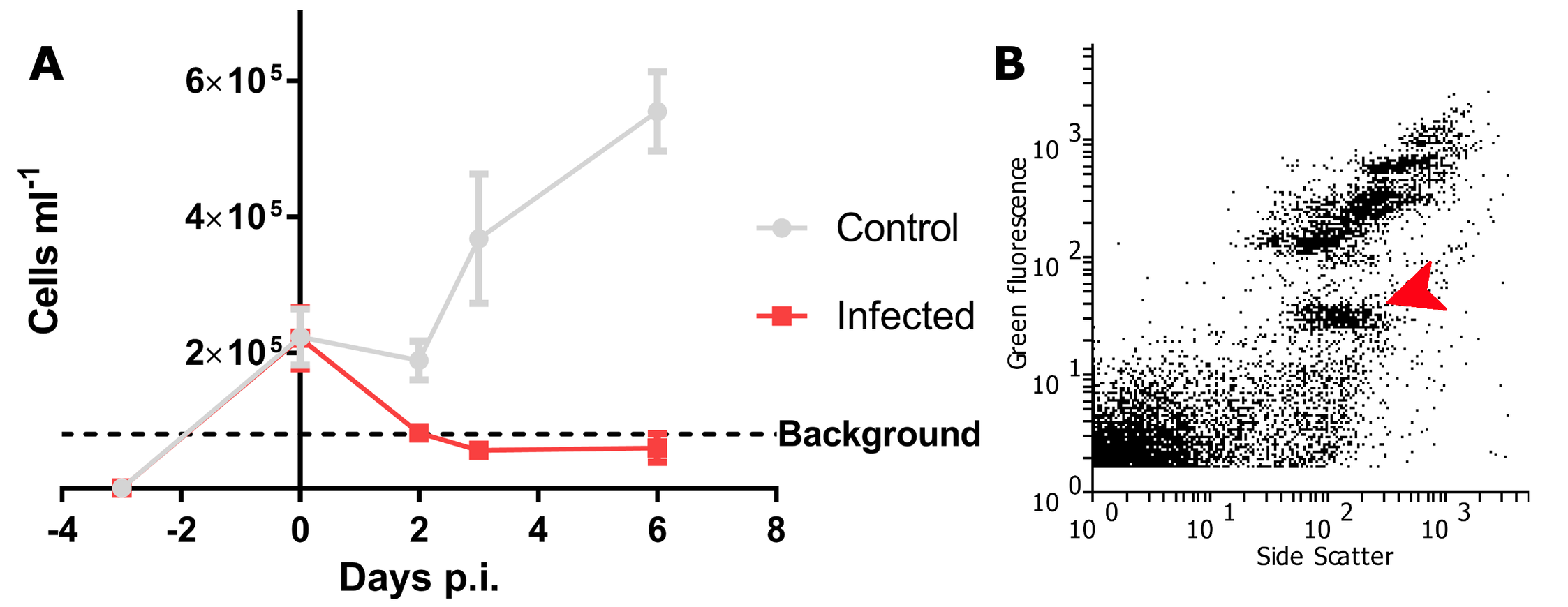

Supplement: S2 Fig — Effect of addition of Chromulinavorax destructans to a culture of Spumella elongata: A: Change in the cell number of S. elongate with (infected) and without (control) exposure to C. destructans added at day 0 post infection (background 8x10^4^ cells ml-1). The error bars represent the SEM of triplicate cultures. B: Flow cytometric profile at 48hpi of C. destructans (red arrow, identified by purification and sequencing) in a culture of S. elongata and stained with SYBR-Green. Chromulinavorax destructans shows a homogeneous genome size (constant green fluorescence signal across the population). Other populations of bacteria with larger genomes (populations with higher green fluorescence levels) are present in the culture and show the typical sloped population indicative of genome replication (higher side scatter correlates with higher green fluorescence). (TIF) [file ppat.1007801.s002.tif]

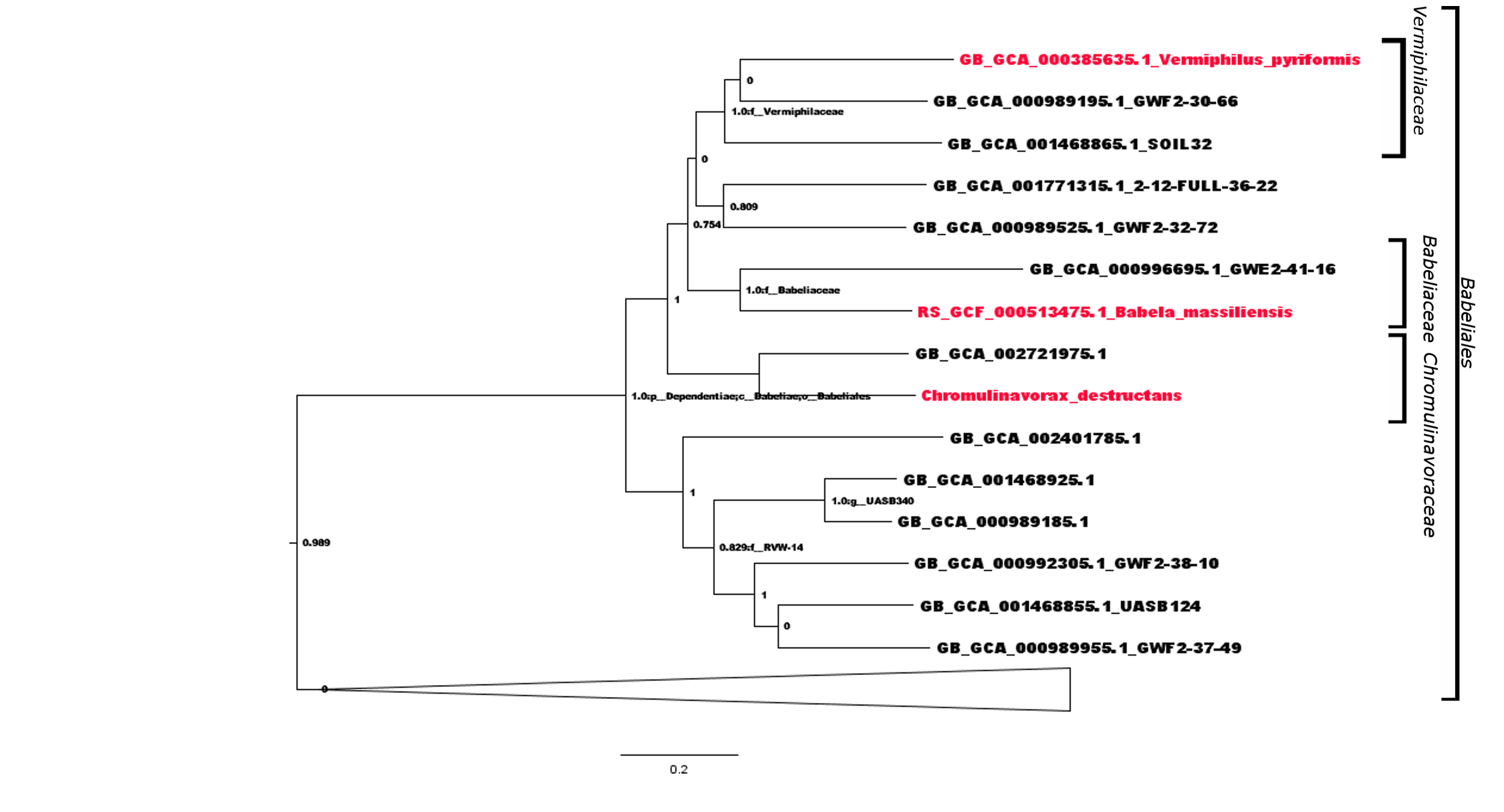

Supplement: S3 Fig — Maximum likelihood tree of the order Babeliales based on genome taxonomy toolkit analysis (Shimodaira-Hasegawa local support values shown; isolates highlighted in red). Chromulinavorax destructans represents a separate family from the Babeliaceae and the recently proposed Vermaphilaceae within this order. The only other genus in the family Chromulinaovoraceae is represented by an incomplete MAG from the South Pacific Ocean. (TIF) [file ppat.1007801.s003.tif]

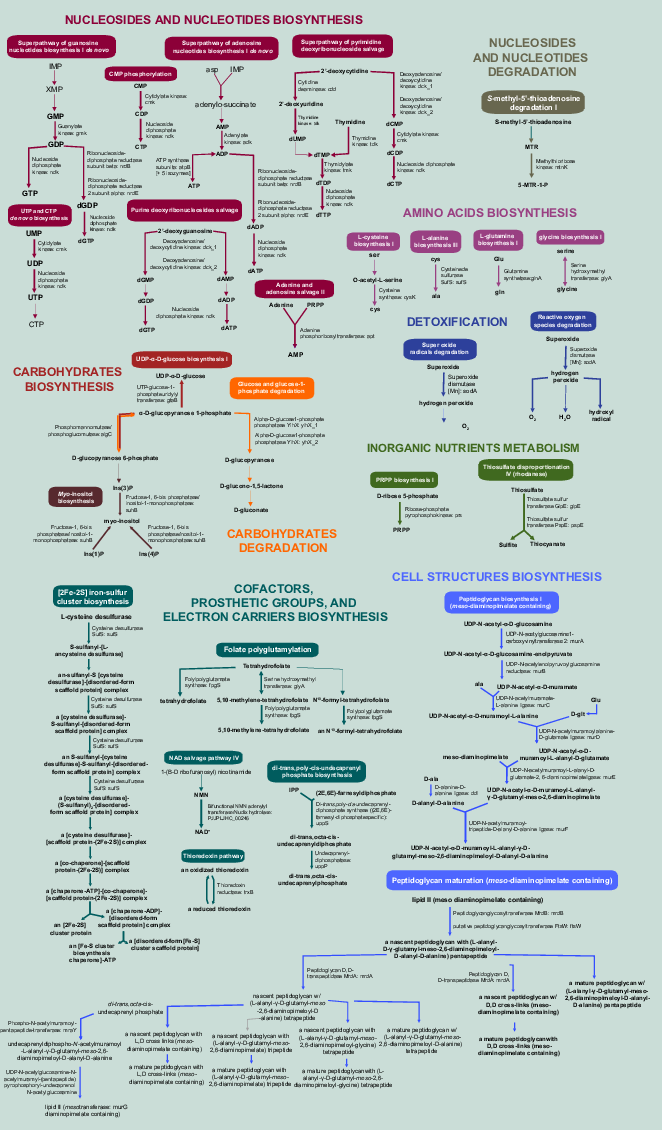

Supplement: S4 Fig — (TIF) [file ppat.1007801.s004.tif]
